# Camp Fever, Alias Scorbutic Fever

**Published:** 1863-02

**Authors:** Alex. McBride


### CAMP FEVER, ALIAS SCORBUTIC FEVER.

By ALEX. McBRIDE, M.D.

I do not propose in this article to give the entire pathology and therapy of camp fever; to do so would require more experience and ability than I have attained to. But as the subject is chiefly important in army practice, and as I have not yet seen any treatise upon it, giving its peculiarities, I think those having had experience with it should promulgate what they know about it, so that such knowledge may be useful while the war lasts, for it is a disease which we will see but little of in civil practice.

*Camp Fever.*—By this designation, I mean a febrile disease which is met with in those persons who have for some weeks or months lived upon the diet and been exposed to the vicissitudes of army life; a fever which in some respects resembles the typhoid fever of Louis, Zenner, Watson and others, the ENTERIC fever of Wood; and which has been so well described by these authors. Tenderness of the abdomen, with tympanitis, and a dry brown tongue, with febrile reaction, and a peculiar diarrhœa, are the group of symptoms, which, with the rose-colored spots, are diagnostic of enteric or typhoid fever.

Camp fever is frequently ushered in with slight chills, frequently repeated for several days, the pulse, generally at least, accelerated from the first. The diarrhœa is full as constant an attendant as in typhoid fever, but tympanitis is neither constant nor frequent. Abdominal tenderness is not so constant as in enteric fever. The tongue is more often large and broad than otherwise, and *always fissured from the first.* The fissures are both *transverse* and *longitudinal* generally, and when the tongue is moist, as it is sometimes, or when it is artificially moistened, the margins of the fissures or cracks have a macerated look, as if penciled with milk and water. This is a very common and peculiar appearance in the advanced stage of the disease. *The fissures are plainly to be seen throughout the whole course of the disease.* It is the first sign of the disease and the last one; it is seen before the patient gives up that he is sick, and it is still to be seen when he is convalescent. These fissures are frequently to be seen in out-door patients who never take to bed with the disease. The cracks of the tongue in enteric fever seem to be dependent on dryness, and are little or not at all perceptible when the tongue is not dry, and disappear entirely as the disease declines.

I have said the fever is frequently ushered in by repeated slight chills. I think this is generally so, but it is difficult to say always how it does begin; for many, if not most cases, have fever tolerably well established when we first see them, especially in the hospitals; but, after having seen the sick soldier in nearly every imaginable condition of camp and field service, I am of the opinion that the inception of what may with propriety be called camp fever is attended with chills. The attending headache is similar to that of enteric fever, but less violent. The urine is diminished in quantity and heightened in density and color, and when the diminution is great there is delirium. This is a circumstance worthy of note. The delirium then would seem to arise from uræmic poisoning, and not from inflammation of intestinal glands, of which there appears to be little or no evidence. The patient generally has appetite for food through the whole course of the fever, and especially for vegetable and acidifying food, and for sour drinks; he not only has appetite, but the food is well borne when prudently prepared and administered. An old physician of much experience in treating fever said, soon after commencing duties in a military hospital, "I never saw men with fever eat so. They eat more like wood-choppers than like sick men." For this reason the semi-liquid stools are copious, and contain the detritus of food as in common diarrhœa.

Now in enteric fever, the patient frequently has some relish for certain kinds of food, from the first, and I believe generally after the first week, but his appetite for it is not strong, neither can he bear much quantity. The kinds best relished and best borne, according to my experience, are *liquid forms of azotized food*, the opposite of that relished and borne in camp fever. But these relishings are probably variable in enteric fever, according to the previous habits or diet of the patient.

In camp fever proper, we do not find the rose-colored spots as in the enteric, but toward the fatal termination we frequently see petechiæ, or what is more like ecchymosis or purpura, and this to a large extent on the trunk and extremities. Alvine evacuations; these are fæcal, more or less mixed with detritus of undigested food; semi-liquid; color not peculiar; odor not peculiar, and this is the case through the whole course of the disease. The quantity is considerable, evidently in consequence of the amount of food taken. This is in striking contrast with the fætid pea-soupy character of the stools of enteric fever, to say nothing of the purulent and briny quality in the ulcerative stage of that disease. The pulse differs in no peculiar respect from that of enteric fever, though I am of the opinion that it is more uniformly soft and somewhat more dull. Latent pneumonia and congestion of the lungs recur probably with nearly the same frequency as in enteric fever. Boils, bed-sores, and cold abscesses also occur. The hair falls out with nearly the same constancy. The termination of this fever in restoration or death depends very much upon the opportunity that may be enjoyed for proper regimen. The febrile type is atonic or typhoid from the beginning, the disease assuming in the advanced stage a very depraved aspect; the patient becoming much emaciated with involuntary evacuations; but although there is a partial stupor, unconsciousness is not complete, the stupidity or inanity resulting not from direct disease or derangement of the nervous centres, but from *lack of animation*.

I am aware that some other febrile disorders occurring in the army are frequently called camp fever. There occurs in the army as much variety of febrile disease as in civil practice, and these occur from the same causes. We meet with every form of inflammatory fever: intermittent, remittent, and bilious fever, also typhoid, and, for aught I know, typhus. I have seen a few cases that resembled typhus more nearly than anything else. They were not enteric, neither did they present the scorbutic character, but were continued fevers of decidedly typhus type. Then we meet with a fever which in the beginning resembles typhoid, except that there is neither tympanitis nor diarrhœa. This fever of continued type will run through a course of two to four weeks without any decided characteristic symptoms, with perhaps an eruption of sudamina, which seem to indicate nothing but abundant warmth and moisture. Similar anomalous fevers are met with in civil practice, and excite no great curiosity.

While among the mountains of Eastern Kentucky, the men were frequently attacked with what we got into the habit of calling mountain ague. It consisted of a seizure, at no very regular intervals, of chills, followed by headache and slight fever without any sweat. Whether this kind of attack was peculiar to that region or whether it is peculiar to an army on a march in a mountainous country, I have not learned. I suppose it was a proper ague. All these fevers are liable to a greater variety of complications than the same diseases met with in civil practice. There is nothing in these fevers or febrile diseases that stamps them as peculiar to the army or camp,—therefore, they should not be embraced within the name of camp fevers. There is no doubt in my mind that the elements of camp fever may combine with the elements of enteric fever and perhaps with other fevers and other diseases, thus producing an extensive variety of effect. It is not in accordance with the plan of this article to offer speculations upon the varieties, especially as I am not positive of their existence.

It would be too much to claim or expect that I should be correct in all the foregoing statements and suggestions with regard to a subject new to the profession at large, and upon which the most favored have but a poor opportuuity to carry out full and complete observations. The most that I expected to accomplish is to set forth distinctly an imperfect model *sui generis*, and hitherto not well distinguished from other continued fevers.

Now, to give in a clear light the diagnostic differences between camp fever and typhoid or enteric fever, I present the following tabular arrangements of those signs and symptoms which are peculiar to each, some of which are available for diagnosis early in the respective diseases.

DIFFERENTIAL SIGNS AND SYMPTOMS.

| CAMP FEVER. | ENTERIC OR TYPHOID FEVER. |
|---|---|
| Abdomen soft, with little or no tenderness. | Abdomen tympanitic, with tenderness more or less intense. |
| Tongue generally large; frequently flabby and pale. | Tongue generally small, and red where not coated. |
| Tongue fissured always; fissures peculiar and continued through the entire course of disease; not dependent on dryness. | Tongue cracked when dry, but fissures not persistent; disappearing when the tongue becomes moist. |
| Appetite generally good, and solid food well borne in considerable quantity, but digestion imperfect. | Appetite slight, and food not borne except in small quantity of liquid form; digestion perfect when too much not taken. |
| No characteristic eruption during any stage of the disease. | Characteristic rose-colored eruption, usually in second or third week. |
| Delirium caused apparently by uremic poisoning. | Delirium caused apparently by enteric inflammation and the general action of fever on sensorial system. |
| Alvine evacuations large and not characteristic. | Evacuations small and characteristic. |

*Pathology and Cause.*—I shall venture nothing positive upon the pathology of camp fever, as I have had almost no opportunity for dissections, for the reasons that there has always been a lack of a proper room at those hospitals where I have had the honor to labor,—these buildings having been constructed in the hurry of military preparation; also there was generally lack of time to make proper investigation; also having regard for the moral effect upon the sick and other soldiers in hospital. These difficulties will be understood by every volunteer army surgeon.

Judging from the appearance of the evacuations, the general absence of tympanitis and tenderness, and the absence of delirium except that obviously caused by uræmic poisoning which sometimes occurs, I am of the opinion that inflammation of the intestinal glands does not belong to this disease. An excited or irritated condition of the mucous membrane of the alimentary canal, caused by the constant passage, in large quantities, of undigested food, and also by the influx of humors and congestion, caused by long continued exposure to cold and damp, is in my opinion the chief pathological condition which, with the scorbutic diathesis, causes this fever. Imperfect digestion and consequent imperfect absorption and assimilation, cause depravity of the fluids and solids of the body. Then, to state the case in brief, the causes of the fever are: Large quantities of azotized food, imperfectly prepared; privation of antiscorbutic food, long continued exposure to low temperature and damp air, the widest possible range of mental and moral excitement and depression. These are the causes which, combined and continued from a few weeks to a few months, produce the peculiar fever. As might be supposed, *a fortiori*, the fever is of vastly more frequent occurrence in new recruits than in old soldiers.

Chemistry does not clearly show us the chemical office of certain kinds of food—the alkalies, the vegetable acids and the acidifying fruits and vegetables; but experience proves their necessity, hence we may call them catalytics. They are necessary to digestion and assimilation, and camp fever arising when these kinds of food are deficient, and never when abundant, hence I conclude that the remote cause of camp fever is lack of catalytic food, with for its development the ordinary causes of diarrhœa superadded. I shall offer no further speculations upon the causes and nature of this disease, but will suggest that, in contradistinction to other fevers which occur in camp, as well as elsewhere, it might with propriety be called *scorbutic fever*.

The camp diarrhœa proceeds, in very many cases, from the same combination of causes,—I mean that diarrhœa which soldiers frequently have without "coming down sick," to use a homely phrase. These cases should be regarded as allied to camp or scorbutic fever, especially those cases having fissured tongues. Cases not presenting any peculiarity of tongue I do not regard as of the same class, and they are curable by ordinary means. I can not let this opportunity pass without speaking of the peculiarity of the tongue presented by many soldiers who have been for some months in the army and undergone the seasoning. The tongue has a cicatrized or *semi-lobulated* appearance on the dorsum, precisely as if many large wounds or sulci had cicatrized. How long this appearance lasts I do not know, neither do I know whether all these cases have had camp fever partially or fully; but from what I have seen in those cases where I knew they had suffered from that disease more or less, I conclude this appearance results from the fissuring caused by that disease.

*Treatment.*—Upon this part of the subject I shall be brief, and set up no claim for dictation, but present a summary of what has been my method. Control the diarrhœa from the beginning through the whole course with opiates, of which class the gum, powder and tincture are the best. The pulv. Doveri has not enough of stimulating quality for so atonic a disease. Tannic acid or ferri persulphas, or tinctura ferri chloridi are necessary and excellent remedies, combined with the opiate in cases where the evacuations are very liquid and copious. When the bowels require unloading, as will sometimes be the case, order thus: ℞. Ol. ricini, flʒ., iv. *vel* fl℥. j; tinct. opii, M. xx. *vel.* xxx. Mix. To be taken at once.

This dose will seldom fail to procure free evacuation in a few hours, and no more active purgation will ever be necessary. Let the opium be continued as a stimulant and anodyne means in dose of about gr. ss. every three to four hours, leaving off the astringent when not indicated. The tinct. ferri, however, may be used sometimes to advantage without reference to any astringent effect.

I have sometimes given in the onset of the disease, where there was little or no diarrhœa: ℞. Quina sulph., Pulv. camphor, aa grains iv., and sometimes one-quarter of a grain of sulphate of morphia added to this. This dose has sometimes appeared to have a very happy effect to mitigate the disease. It appears to generalize the circulation and thereby mitigate the fever and diarrhœa; but this dose will produce no marked salutary effect after the disease is established fully.

Spirits mindereri, with the acid in excess in the dose fl℥. ss. to j., taken in conjunction or alternation with the opiate, is a most excellent remedy to promote moisture of the mouth and skin. It abates the thirst, and in my opinion facilitates the urinary secretion. I suppose almost any physician who had treated typhoid enteric fever would expect in the disease in question to effect moisture of the dry tongue by the exhibition of turpentine emulsion, but in this he would be disappointed. With this medicine the tongue will become more dry and the case will not improve, and this fact seems to mark a difference in the pathology of this from enteric fever. Wood says in his excellent treatise on enteric fever (vide *Wood's Practice of Medicine*, vol. i., p. 357) : "I can not too strongly impress upon the profession my convictions of the importance of this medicine" (turpentine). "It may be employed in all cases in the advanced stages of this disease when the tongue is dry. But there is a particular condition, in which I have very often employed it, and hitherto have seldom known it fail." Then he further goes on in clear and forcible terms to show that this "condition" is that of ulceration. This part of Wood's treatise is all important to those who would successfully treat enteric fever, and my experience has shown to me that it is important to make the distinction in treating camp fever. This fact, in default of other evidence, would seem to indicate that the pathology of the two fevers is different.

The partial suppression of urine, which is perhaps the most fatal incident of this disease, does not seem to me to be an indication for turpentine, even though the mouth is parched at the same time. In this state of the case acetate of potassa is a good remedy, and a very convenient form in the camp is: ℞. Bicarb. potass., ℈j.; common vinegar, water, aa fl℥ ss. M. Take effervescing. Repeat every three to four hours. Nitrous ether also may be given to advantage. In these cases the patient is delirious, and the prognosis should be unfavorable. In this feature of the disease, as in the disease generally, vegetable acid drinks should be freely given, of which vinegar and water is as good as any, and very grateful to the patient.

Mercury as a remedy in this disease requires no further mention than that of condemnation. It has no place in the treatment.

Epispastics should be used to fulfil indications as in other diseases. In the congestion and inflammation of the lungs, which sometimes complicate this disease, *blistering ought not to be omitted*, and it should be repeated as often as indicated. Blistering for the delirium of uræmic poison is of doubtful efficacy. If applied at all, I think it should be on the lower parts of the trunk or to the thighs, with the view of re-establishing the renal secretion.

I shall add nothing further on the medical treatment of this disease. Its various complications and phases are to be managed on rational principles, keeping the peculiarity of the disease in view. Regimen is everything in this disease; I mean that proper regimen is paramount to everything else. Cleanliness, quiet, and proper warmth, with free ventilation, demand the physician's first care. The food should consist of well prepared common food, with a considerable preponderance of the anti-scorbutic vegetables. I have seen patients who were quite low with this disease, eat boiled potatoes and carefully and finely cut cold slaw. They ate such food with a relish and to advantage. Cabbage soup, potatoe soup, boiled turnip with butter, milk sweet or sour, according to taste, etc., etc.; and the patient's appetite, which is generally pretty good, should be indulged to a considerable extent. Acid drinks are important, and ought not to be neglected. Hard cider and native wine properly diluted are perhaps the best, but in field practice these can seldom be had in sufficient quantity; but fortunately the commissariat generally supplies abundance of vinegar, and I am of the opinion, after considerable experience in its use, that it is as good, if not better than any other vegetable acid. Let it be drank freely by all the patients who relish it, and this will be nearly all, diluted with eight to ten parts of water to one of vinegar; and this can be varied by sometimes adding a little sugar, and sometimes to this again a little whiskey. Citric and tartaric acids can also be used for variety, and when these are scarce, cream of tartar can be substituted. The jellies and sour fruits now so much in vogue, and so munificently furnished by the various sanitary societies, are valuable in the nourishing of these patients.

I am very much of the opinion that a regimen similar to the above, diligently and carefully pursued from the onset, would alone be sufficient for the recovery of most cases of scorbutic or camp fever.

If in the foregoing rude attempt to briefly portray camp fever as my experience has impressed it upon my mind, I have shed some light, and will aid thereby in calling out other facts upon the subject, the reader's time will not have been lost nor will my labor have been in vain.—*Lancet and Observer*.

---

American Medical Monthly.—It is announced in the December number of this journal that its publication will be suspended.—*Medical News and Library*.

